# Physical Exercise-Induced Astrocytic Neuroprotection and Cognitive Improvement Through Primary Cilia and Mitogen-Activated Protein Kinases Pathway in Rats With Chronic Cerebral Hypoperfusion

**DOI:** 10.3389/fnagi.2022.866336

**Published:** 2022-06-01

**Authors:** Wenyue Cao, Junbin Lin, Wei Xiang, Jingying Liu, Biru Wang, Weijing Liao, Ting Jiang

**Affiliations:** ^1^Department of Neurorehabilitation, Zhongnan Hospital of Wuhan University, Wuhan University, Wuhan, China; ^2^Department of Orthopedics, Renmin Hospital of Wuhan University, Wuhan University, Wuhan, China

**Keywords:** physical exercise, primary cilia, astrocytes polarization, MAPKs pathway, cognition, chronic cerebral hypoperfusion

## Abstract

Chronic cerebral hypoperfusion (CCH) is closely related to vascular cognitive impairment and dementia (VCID) and Alzheimer’s disease (AD). The neuroinflammation involving astrocytes is an important pathogenic mechanism. Along with the advancement of the concept and technology of astrocytic biology, the astrocytes have been increasingly regarded as the key contributors to neurological diseases. It is well known that physical exercise can improve cognitive function. As a safe and effective non-drug treatment, physical exercise has attracted continuous interests in neurological research. In this study, we explored the effects of physical exercise on the response of reactive astrocytes, and its role and mechanism in CCH-induced cognitive impairment. A rat CCH model was established by 2 vessel occlusion (2VO) and the wheel running exercise was used as the intervention. The cognitive function of rats was evaluated by morris water maze and novel object recognition test. The phenotypic polarization and the primary cilia expression of astrocytes were detected by immunofluorescence staining. The activation of MAPKs cascades, including ERK, JNK, and P38 signaling pathways, were detected by western blot. The results showed that physical exercise improved cognitive function of rats 2 months after 2VO, reduced the number of C3/GFAP-positive neurotoxic astrocytes, promoted the expression of S100A10/GFAP-positive neuroprotective astrocytes, and enhanced primary ciliogenesis. Additionally, physical exercise also alleviated the phosphorylation of ERK and JNK proteins induced by CCH. These results indicate that physical exercise can improve the cognitive function of rats with CCH possible by promoting primary ciliogenesis and neuroprotective function of astrocytes. The MAPKs signaling cascade, especially ERK and JNK signaling pathways may be involved in this process.

## Introduction

Vascular diseases are always related to the cognitive impairment (Duncombe et al., 2017; Tang et al., 2021; Ungvari et al., 2021). More and more evidence show that vascular risk factors can lead to neurodegeneration, cognitive impairment and dementia (Duncombe et al., 2017). Certain areas of the brain responsible for the memory, cognition, and behavior are particularly vulnerable to insufficient blood supply (Thammisetty et al., 2021). Chronic cerebral hypoperfusion (CCH) often occurs due to diseases that affect the cerebral circulatory system, such as hypertension, diabetes, atherosclerosis and smoking, and is one of the prime factors leading to the development of vascular cognitive impairment and dementia (VCID) in the elderly (Thammisetty et al., 2021). CCH is also considered as a preclinical condition of mild cognitive impairment and precursor of dementia (Xu et al., 2020). In addition, CCH is a common and important cause of cognitive impairment associated with many neurodegenerative diseases, such as Alzheimer’s disease (AD) and Parkinson’s disease, and it plays an indispensable role in the development of these diseases (Tang et al., 2017; Xie et al., 2018; Feng et al., 2021). Recent studies conducted by the American AD Research Center revealed that up to 80% of the sporadic and late onset AD have some form of brain vascular pathology (Duncombe et al., 2017; Xu et al., 2020). The inflammation, oxidative stress, and amyloid-β (Aβ) accumulation commonly existed in AD pathology have been reported to be associated with CCH. In addition, CCH was found to appear in the “early” stage of AD. Before the obvious symptoms of AD appear, arterial spin-labeled magnetic resonance imaging has detected changes in cerebral blood flow, indicating that CCH may be a potential biomarker of AD (Duncombe et al., 2017; Xu et al., 2020). In general, it has been demonstrated that there is considerable overlap between the characteristics of VCID and AD. CCH seems to be the common underlying pathophysiological mechanisms, and it is the main contributor to cognitive decline and degenerative development of dementia (Duncombe et al., 2017; Xu et al., 2020).

It is well known that neuroinflammation is considered to be the important pathogenic mechanism of CCH-induced cognitive impairment (Du et al., 2020). Considerable evidence shows that CCH can promote the occurrence and progression of cognitive impairment and dementia through neuroinflammation (Kim et al., 2017). Astrocytes are the most abundant cells and play multiple roles in the central nervous system (CNS). It has been confirmed that astrocytes participate in the immune response of the CNS, and are potential sentinels in the brain parenchyma together with microglia (Leardini-Tristao et al., 2020). In addition, astrocytes are not only involved in the regulation of neurotransmission, neurodevelopment, cerebral blood flow, metabolism and neurogenesis, their coverage integrity but also related to the brain homeostasis and cognition (Leardini-Tristao et al., 2020; Miyamoto et al., 2020). Recent studies have confirmed that astrocytes have two phenotypes, the neurotoxic A1 and neuroprotective A2 phenotype (Liddelow et al., 2017). However, astrocytes activated after CCH mainly transform to A1 phenotype, which lose their normal function and aggravate the neuroinflammation progression (Miyamoto et al., 2020; Jiang et al., 2021). On the one hand, the loss of normal A1 astrocytes function and the interruption of their interaction with blood vessels may lead to homeostasis destruction of neurovascular units and cognitive impairment (Leardini-Tristao et al., 2020; Presa et al., 2020). On the other hand, it has been shown that the neuroinflammatory processes aggravated by the activated neurotoxic astrocytes are essential for the initiation and progression of cognitive disorders (Kim et al., 2017). Therefore, modulating the polarization of activated astrocytes from neurotoxicity to neuroprotective phenotype may be one of the potential therapeutic targets for CCH-induced cognitive impairment.

There is evidence that primary cilia play important roles in regulating inflammation, and the disturbance of primary cilia may lead to gliosis and neuroinflammation (Singh et al., 2019). Primary cilia are essential for the development and maintenance of neural homeostasis in the mammalian brain (Sipos et al., 2018). They act as the “sensing antenna” of cells and are the central hub for receiving and transducing extracellular signals to elaborate biological responses to a wide range of developmental and physiological processes. Primary cilia always play key roles in cell cycle control, cell proliferation, migration, and polarity (Alvarez-Satta et al., 2019; Alhassen et al., 2021). Additionally, large numbers of convincing studies have shown that there are strong correlations between primary cilia and cognition (Hu et al., 2017; Alhassen et al., 2021). Defects in primary cilia can lead to age-related cognitive decline, and primary cilia may be potential targets for AD and/or other dementia (Alvarez-Satta et al., 2019). Most mammalian cells have primary cilia, including astrocytes (Sipos et al., 2018). In response to various brain injuries, such as traumatic brain injury, ischemia, and neurodegenerative diseases, astrocytes proliferation is coordinated with the ciliogenesis and dismantling of astrocytes cilia (Sterpka and Chen, 2018). A study has reported that primary cilia dysfunction caused by congenital deletion of BBSome protein 8 can induce A1-like astrocyte activation, neuroinflammation, and changes in postsynaptic density (Singh et al., 2019). Primary cilia are an important component of the sonic hedgehog (Shh) signaling pathway, and the activation of the latter can promote astrocyte polarization toward to A2 phenotype (Payan-Gomez et al., 2018). These studies indicate that primary cilia are involved in the regulation of the A1/A2 phenotypic polarization of astrocytes. At present, little is known about the physiological and pathological functions of astrocytic primary cilia (Sterpka et al., 2020). Further exploration of the role of primary cilia in reactive astrocytes will not only help to better understand the regulatory mechanism of astrocytes polarization, but also to develop potential therapeutics for cognitive disorders.

Over the years, there have been many studies on the benefits of physical exercise. A lot of scientific evidences show that there is a positive correlation between active sports lifestyle and health benefits (De la Rosa et al., 2020; Marques-Aleixo et al., 2021; Padilha et al., 2021). Physical exercise is an effective non-toxic strategy to prevent and treat many chronic diseases. Its positive effects in reducing the risk of all-cause death and extending life span have been widely documented (Scheffer and Latini, 2020). More and more evidences showed that physical exercise, including aerobic and resistance exercise training, can alleviate age-related cognitive impairment (Brach et al., 2021; Chow et al., 2021; Pronk, 2021). Physical exercise can promote different physiological phenomena including neurogenesis, synaptogenesis, angiogenesis, and neurotrophic factor stimulation by inducing a series of cellular and molecular processes, thereby causing changes in the brain at the cellular, molecular and anatomic level, and eventually enhancing learning, memory and brain plasticity (De la Rosa et al., 2020). Additionally, the beneficial effects of physical exercise on health outcomes also involve the regulation of the immune system. Regular physical exercise can prevent viral and bacterial infections and enhance the immune response to vaccines and pathogens. This makes physical exercise a valuable tool for preventing infectious diseases such as COVID-19 (Scheffer and Latini, 2020). Moreover, studies have shown that physical inactivity favors promoting the polarization of immune cells toward a pro-inflammatory phenotype. In contrast, regular moderate-intensity physical exercise can direct the immune response to an anti-inflammatory state, which is considered to be the main molecular mechanism required for the improvement of health outcomes (Scheffer and Latini, 2020). Our previous study also found that physical exercise can regulate the polarization of astrocytes from pro-inflammatory A1 to neuroprotective A2 phenotype, and improve the cognitive impairment of rats with CCH (Jiang et al., 2021).

In this study, we will investigate the effects of physical exercise on primary ciliogenesis and astrocyte polarization in rats with CCH. In addition, we will further investigate the role of MAPKs signaling cascades, including ERK, JNK, and P38 signaling pathways in this process.

## Materials and Methods

### Experimental Animals and Groups

**FIGURE 1:**
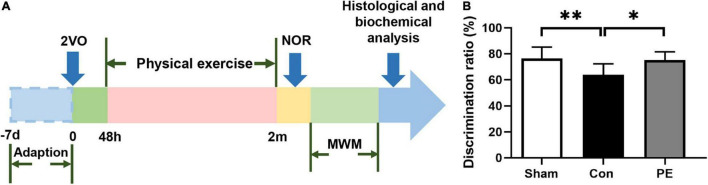
Flow diagram of the experiment and the novel object discrimination ratio in rats with chronic cerebral hypoperfusion. **(A)** Flow chart of experimental design to explore the effect and mechanism of physical exercise on cognitive function in 2VO rats. 2VO, 2 vessel occlusion; NOR, novel object recognition; MWM, Morris water maze. **(B)** The novel object discrimination ratio of rats in each group. *n* = 10. Experimental data are expressed as the means ± SD. *P < 0.05, ^**^P < 0.01. Con, control; PE, physical exercise.

In this experiment, male Wistar rats 280–320 g were used and purchased from Unilever Laboratory Animal Company, Beijing. The rats were kept in cages with ambient temperature of 23 ± 1°C, humidity of 55 ± 5%, light/dark cycle of 12 h, and free access to food and water. All animal experiments were conducted in accordance with the Guide for the Care and Use of laboratory Animals of the National Institutes of Health (Publication No. 80-23, revised 1996) and approved by Institutional Animal Care and Use Committee of Wuhan University Center for Animal Experiment. The experimental rats were randomly divided into sham group and 2-vessel occlusion (2VO) group. The successful 2VO rats were further divided into control group and physical exercise group randomly. The experimental procedure is shown in Figure 1A.

### Experimental Animal Model

Chronic cerebral hypoperfusion model was established by bilateral common carotid artery occlusion (2VO) in rats as previously described. For the 2VO operation, the rats were anesthetized with pentobarbital and the neck was incised longitudinally through the midline. Subsequently, the bilaterally common carotid arteries were exposed and carefully separated from the adjacent vagus nerve. The distal and proximal portions of the common carotid arteries were double permanently ligated with two 4-0 silk wires, respectively. Finally, the bilaterally common carotid arteries were cut between the middle of the two ligations. Rats in the sham group were subjected to the same procedure without ligation and cutting. The rats were placed on the heating pad and observed until they were fully awake before being brought back to the cage.

### Physical Exercise Intervention

As described in our previous study (Jiang et al., 2017, 2021), rats in the physical exercise group were given electric wheel running training 48 h after 2VO. The initial speed was 5 rpm and gradually increased to 7 rpm in the first week after 2VO. It increased to 10 rpm in the second week, 15 rpm in the third week, 20 rpm in the fourth week after 2VO, and then maintained this speed until behavioral testing. The training time is 20 mins each time, two times a day, and six consecutive days a week. The animals in sham group and control group were kept in cages without exercise training intervention.

### Morris Water Maze Test

The water maze test was used to evaluate the hippocampus-dependent spatial learning and memory of rats (Ghafarimoghadam et al., 2022). The experiment was carried out in a circular tank with a water depth of 30 cm, a diameter of 150 cm, and a height of 50 cm. The pool was divided into four quadrants, and a 1.5 cm underwater platform was placed at a fixed location in one of the quadrants. The place navigation test was designed for 5 days and four times a day. The animals were released into the water from a position in the middle of the outer edges of the four quadrants and facing the wall of the pool. During each trial, the escape latency was recorded as the time to find the platform within 60 s, and rats that could not find the platform within 60 s were guided to the platform. After the 5 day place navigation test, the underwater platform was taken away, and a spatial probe test was conducted on each animal to evaluate the reference memory. The data was recorded by Animal Video Tracking Analysis System (Anilab Scientific Instruments Co., Ltd., Ningbo, China).

### Novel Object Recognition Test

The novel object recognition test was used to evaluate non-spatial memory between the prefrontal and subcortical circuits in rats. As described in previous studies (Jiang et al., 2017, 2021), the experiment was carried out in a transparent open box (72 cm^3^ × 72 cm^3^ × 35 cm^3^). On the day before the test, rats were allowed to explore the box freely without any objects for 10 mins. The test on the second day was divided into two phases: familiarization and testing phase. In the familiarization phase, rats were placed in the box containing two identical objects and explored freely for 5 mins, and were considered to be exploring when they touched the objects with their noses or were within 1 cm of the objects. After the familiarization period, the box and objects were cleaned with 70% ethanol to eliminate residual odors. One hour later, during the testing phase, one of the objects was replaced with a new object of completely different size and shape, and the rats were allowed to explore the two different objects for 5 mins. The exploration time of familiar object (F) and new object (N) were recorded, respectively, and the discrimination ratio (DR) = N/(N + F) was calculated × 100%.

### Tissue Preparation for Histochemistry

Two months after 2VO, rats in each group were sacrificed after behavioral testing. Rats were deeply anesthetized with pentobarbital and perfused transcardially with 0.9% saline at 4°C and 4% paraformaldehyde (PFA) successively. The brain tissues of rats were quickly separated and fixed in 4% PFA at 4°C for 24 h, and then dehydrated in 20% and 30% sucrose. Then, the brain tissues were embedded with OCT (American cherry blossom) and placed in a cryomicrotome (CM1900, Leica, Germany) for continuous coronal sectioning (10 μm) for subsequent histological experiments.

### Immunofluorescence Staining

Immunofluorescence staining was as previously described (Jiang et al., 2017, 2021), the brain sections were pretreated in citrate buffer (85°C) for 5 mins for antigen retrieval, blocked with immunostaining blocking buffer (P0102, Beyotime, China) for 1 h at room temperature and then incubated overnight at 4°C with the following primary antibody: mixtures of mouse anti-GFAP antibody (1:200, Boster, China) and goat anti-C3d antibody (1:200, R&D, United States), mouse anti-GFAP (1:200, Boster, China) and chicken anti-S100A10 antibody (1:200, Abcam, United Kingdom), mouse anti-GFAP (1:200, Boster, China) and rabbit anti-ARL13B antibody (1:200, Proteintech, United States). The following day, brain sections were incubated with mixtures of goat anti-mouse IgG (DyLight 488 Conjugated; 1:200, Boster, China) and rabbit anti-goat IgG (CY3 Conjugated AffiniPure; 1:200, Boster, China), goat anti-mouse IgG (DyLight 488 Conjugated; 1:200, Boster, China) and goat anti-chicken IgG (Goat Anti-Chicken IgY; 1:400, Abcam, United Kingdom), and goat anti-mouse IgG (DyLight 488 Conjugated; 1:200, Boster, China) and goat anti-rabbit IgG (Alexa Fluor^®^ 555 Conjugate; 1:500, Cell Signaling Technology, Boston, MA, United States) for 1 h at room temperature. Then, sections were washed with phosphate buffered solution (PBS) and sealed with a DAPI-containing antifade solution. The fluorescence signals were examined by fluorescence microscopy (BX53; Olympus).

### Western Blot Analysis

The total protein was extracted from the corpus callosum of rats for western blot analysis. Briefly, rats were deeply anesthetized with pentobarbital and perfused transcardially with 4°C saline (50 ml). The corpus callosum was rapidly separated and removed on ice. After adding the lysis buffer, the brain tissue was fragmented using a tissue grinder. Protein concentrations of all extracted samples were measured using Bio-Rad Protein Assay (BioRad, Hercules, CA, United States) and bovine serum albumin (BSA) standards. A 30 μg protein sample was loaded and separated by sodium dodecyl sulfate-polyacrylamide gels (SDS-PAGE) and transferred to a polyvinylidene fluoride (PVDF) membrane, which was then incubated overnight at 4°C with the following primary antibody: ERK (1:1,000, Cell Signaling Technology, Boston, MA, United States), P-ERK (1:1,000, Beyotime, China), JNK (1:1,000, Cell Signaling Technology, Boston, MA, United States), P-JNK (1:1,000, Beyotime, China), P38 (1:1,000, Cell Signaling Technology, Boston, MA, United States), P-P38 (1:1,000, Beyotime, China) and GAPDH (1:1,000, Servicebio, Wuhan, China). Next, the membrane was washed and incubated with horse-radish peroxidase-labeled goat anti-rabbit and goat anti-mouse secondary antibody (1:10,000, Boster, China) for 1 h at room temperature. Finally, the enhanced chemiluminescence system was used to observe protein bands.

### Statistical Analysis

Statistical analysis was performed using IBM SPSS Statistics 20.0 and GraphPad Prism 8.0. The data are expressed as mean ± SD. In the behavioral tests, the movement trajectories and data are recorded by behavioral recording software (Anilab Scientific Instruments Co., Ltd., Ningbo, China). For immunofluorescence, three slices of each brain were selected at the corpus callosum, and 3–6 regions of each slice were randomly selected to be photographed for analysis. To measure the length of primary cilia, the standard scale bar was used as the reference and the length of primary cilia was measured from the base to the top by Image J software (National Institutes of Health, Bethesda, MD, United States). All immunofluorescence data were analyzed by Image J software. For western blot, the gray value of the western blot bands was measured by Image J software, and the gray value of the target protein was compared with the GADPH. The escape latency and swimming speed in the MWM test were analyzed by repeated measures analysis of variance (ANOVA). The platform crossings and dwell time in the MWM test, the discrimination index in the NOR test and the results of immunofluorescence and Western blotting were evaluated by one-way ANOVA followed by Tukey’s *post hoc* test. *P* < 0.05 is considered to be statistically significant.

## Results

### Physical Exercise Improved Cognitive Function in Rats With Chronic Cerebral Hypoperfusion

**FIGURE 2:**
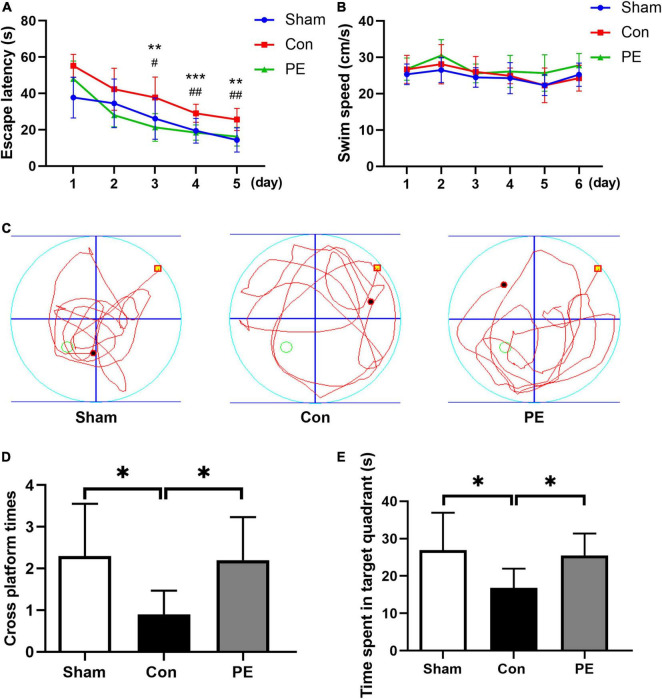
Physical exercise improved spatial memory in rats with chronic cerebral hypoperfusion. In the place navigation test: **(A)** the escape latency of rats in each group. PE vs. Con: **P < 0.01, ***P < 0.001; Sham vs. Con: *^#^P <* 0.05, *^##^P <* 0.01. **(B)** The swimming speed of rats in each group. There was significant difference in the escape latency (*P* < 0.001) but not the swimming speed (*P* > 0.05) between the different groups in the overall. In the spatial probe test: **(C)** Representative swimming trajectories of rats in each group. **(D)** Times of crossing the target platform of rats in each group. **(E)** Time spent in the target quadrant of rats in each group. *n* = 10. Experimental data are expressed as the means ± SD. *P < 0.05, ^**^P < 0.01. Con, control; PE, physical exercise.

Spatial and non-spatial learning memory abilities of rats 2 months after 2VO were assessed by MWM and NOR test, respectively. The results of the NOR test showed that the ability of novel object recognition in the control group was impaired compared to the sham group (63.91 ± 8.44 vs 76.29 ± 8.83%; *P* < 0.01). However, physical exercise alleviated this deficit as shown by the significantly improved discrimination index for novel objects (75.13 ± 6.44 vs 63.91 ± 8.44%; *P* < 0.05; Figure 1B). The results of the MWM test showed that in the place navigation test (Figures 2A,B), the control group had a longer escape latency to find the underwater platform on days 3–5 compared to the sham group (day 3: 37.74 ± 11.28 vs 26.17 ± 11.37 s, *P* < 0.05; day 4: 29.04 ± 4.96 vs 19.47 ± 6.79 s, *P* < 0.01; day 5: 25.67 ± 6.08 vs 14.38 ± 6.66 s, *P* < 0.01; respectively). However, the escape latency of the physical exercise group on days 3–5 was significantly shorter than the control group (day 3: 21.28 ± 7.56 vs 37.74 ± 11.28 s, *P* < 0.01; day 4: 18.46 ± 4.18 vs 29.04 ± 4.96 s, *P* < 0.001; day 5: 16.19 ± 5.11 vs 25.67 ± 6.08 s, *P* < 0.01; respectively). In the spatial probe test (Figures 2C–E), the control group had a reduced number of platform crossings (0.90 ± 0.57 vs 2.30 ± 1.25, *P* < 0.05) and decreased dwell time in the target quadrant (16.81 ± 5.17 vs 26.94 ± 10.00 s, *P* < 0.05) compared with the sham group. However, physical exercise increased the number of crossing the platform (2.20 ± 1.03 vs 0.90 ± 0.57, *P* < 0.05) and the time spent in the target quadrant (25.47 ± 5.91 vs 16.81 ± 5.17 s, *P* < 0.05) in rats relative to the control group. The above results indicate that physical exercise can improve not only the spatial memory but also the non-spatial memory between the prefrontal and subcortical circuits in rats with CCH.

### Physical Exercise Promoted the Polarization of Astrocytes to Neuroprotective Phenotype

**FIGURE 3:**
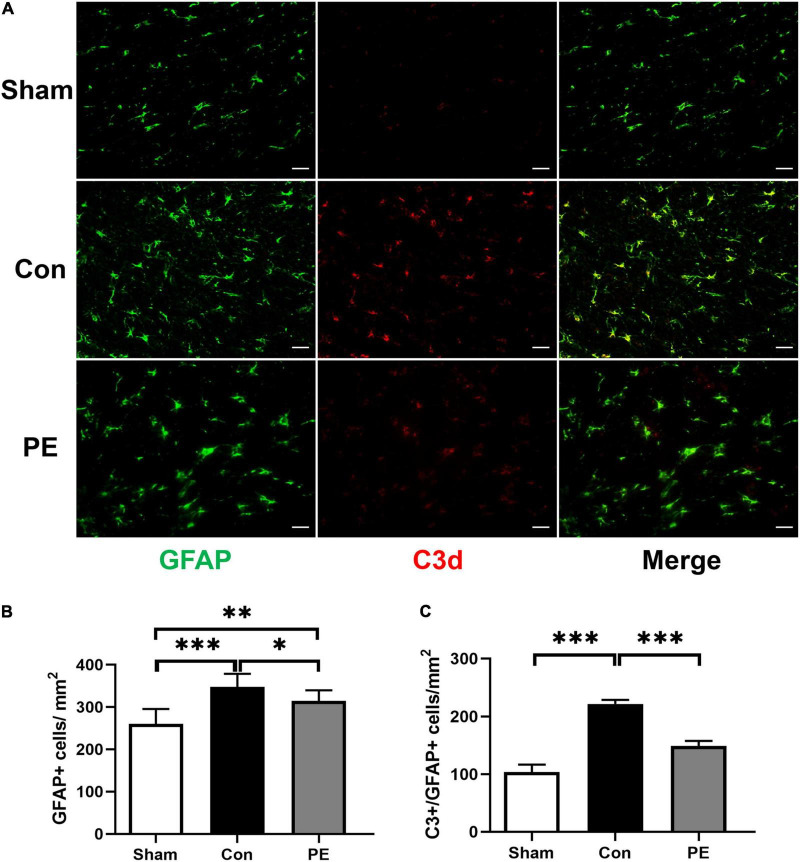
Physical exercise decreased the number of A1 neurotoxic astrocytes. **(A)** Representative immunofluorescence images for GFAP (green) and C3 (red) double staining of rats in each group in the corpus callosum. Scale bar = 50 μm. **(B)** The quantification of GFAP positive astrocytes and **(C)** C3/GFAP positive A1 astrocytes. *n* = 6. Experimental data are expressed as the means ± SD. *P < 0.05, ^**^P < 0.01, ^***^P < 0.001. GFAP, glial fibrillary acidic protein; C3, complement 3.

**FIGURE 4:**
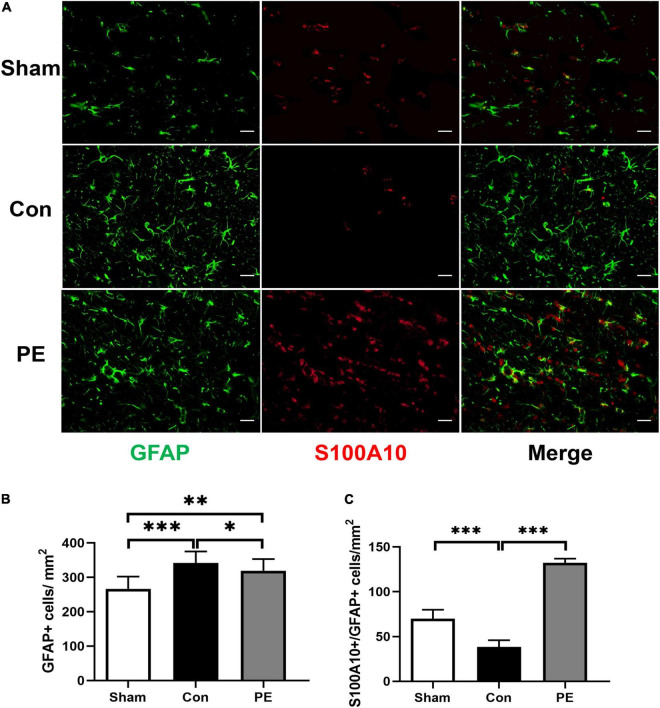
Physical exercise enhanced the expression of A2 neuroprotective astrocytes. **(A)** Representative immunofluorescence images for GFAP (green) and S100A10 (red) double staining of rats in each group in the corpus callosum. Scale bar = 50 μm. **(B)** The quantification of GFAP positive astrocytes and **(C)** S100A10/GFAP positive A2 astrocytes. *n* = 6. Experimental data are expressed as the means ± SD. *P < 0.05, ^**^P < 0.01, ^***^P < 0.001. S100A10, S100 calcium binding protein A10.

To investigate the effect of physical exercise on the phenotypic polarization of astrocytes in the brain of rats 2 months after 2VO, the C3/GFAP and the S100A10/GFAP immunofluorescent double-label staining were performed. In which C3 is considered as a marker for neurotoxic astrocytes of A1- phenotype and S100A10 is considered as a marker for neuroprotective astrocytes of A2- phenotype. The results showed an increased number of GFAP-positive astrocytes in the control group compared with the sham group (*P* < 0.001; Figures 3A,B, 4A,B), indicating that there is astrocytes response and activation after CCH. At the same time, the number of C3/GFAP-positive astrocytes was also significantly higher in the control group (221.63 ± 7.14 vs 103.45 ± 13.02/mm^2^; *P* < 0.001; Figures 3A,C), suggesting that activated astrocytes may shift primarily toward the A1 phenotype. However, physical exercise reduced the number of C3/GFAP-positive astrocytes (149.00 ± 8.41 vs 221.63 ± 7.14/mm^2^; *P* < 0.001) and promoted the expression of S100A10/GFAP-positive astrocytes (132.13 ± 4.75 vs 38.41 ± 7.37/mm^2^; *P* < 0.001) compared to the control group (Figures 3A,C, 4A,C). These results suggest that physical exercise may create a favorable microenvironment for promoting the activated astrocytes polarization from A1 to A2 phenotype, and eventually exert neuroprotective functions.

### Physical Exercise Enhanced Primary Cilia Expression and Promoted the Length Recovery of Primary Cilia in Astrocytes

**FIGURE 5:**
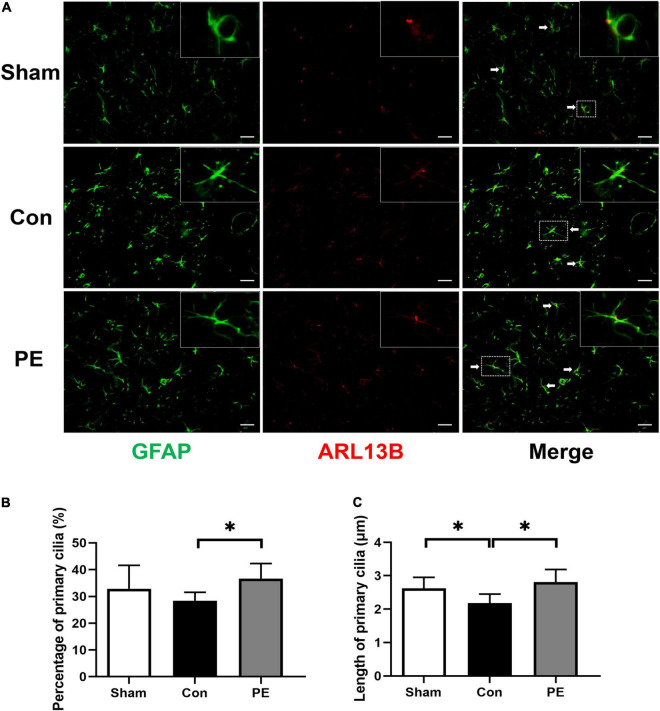
Physical exercise augmented the expression and length of primary cilia in astrocytes. **(A)** Representative immunofluorescence images for GFAP (green) and ARL13B (red) double staining of rats in each group in the corpus callosum. Scale bar = 50 μm. **(B)** Quantification of the percentage of astrocytes containing primary cilia. **(C)** Quantification of length of astrocytic primary cilia. *n* = 6. Experimental data are expressed as the means ± SD. *P < 0.05. ARL13B, ADP-ribosylation factor-like 13B.

The ARL13B/GFAP immunofluorescence double staining was performed to evaluate the effects of physical exercise on primary cilia expression in astrocytes of rats after 2VO. ARL13B is considered to be the main marker of primary cilia in astrocytes. The results showed that the average length of astrocytic primary cilia in the sham group was about 2.62 ± 0.33 μm (Figure 5C). Compared with the sham group, the expression (28.39 ± 3.17 vs 32.86 ± 8.76%; *P* > 0.05) and length of primary cilia (2.18 ± 0.27 vs 2.62 ± 0.33 μm; *P* < 0.05) in astrocytes were decreased in the control group (Figures 5A–C). The results indicate that the primary cilia of astrocytes have undergone adaptive expression and morphological changes to reduce the cilia signal transduction in astrocytes, in turn affecting astrocytes responses and functions to injury under CCH. However, physical exercise can promote not only astrocytic primary ciliogenesis (36.69 ± 5.59 vs 28.39 ± 3.17%; *P* < 0.05; Figures 5A,B), but also the restoration of primary ciliary length (2.81 ± 0.38 vs 2.18 ± 0.27 μm; *P* < 0.05; Figures 5A,C). These results suggest that physical exercise may affect the function of astrocytes by regulating the occurrence and length of astrocytic primary cilia.

### Physical Exercise Reduced the Phosphorylation Levels of ERK and JNK Pathway

**FIGURE 6:**
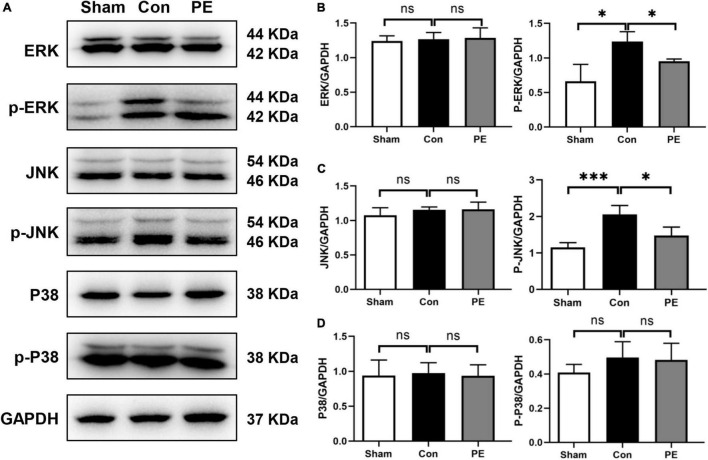
Physical exercise inhibited ERK and JNK phosphorylation. **(A)** Representative images for western blotting of ERK, p-ERK, JNK, p-JNK, P38, and p-P38 expressions. **(B–D)** Densitometry analyses of the expression of ERK, p-ERK, JNK, p-JNK, P38, and p-P38 normalized to GAPDH. *n* = 5. Experimental data are expressed as the means ± SD. *P < 0.05, ^***^P < 0.001.

To assess the effects of physical exercise on the MAPKs signaling cascades, including ERK, JNK and P38 signaling pathways in rats 2 months after 2VO, Western blot analysis was performed to detect the protein expression of ERK1/2, P-ERK1/2, JNK, P-JNK, P38, and P-P38 in the corpus callosum. According to the results, the control group showed increased phosphorylation level of ERK1/2 and JNK compared with the sham group (*P* < 0.05 and *P* < 0.001). However, physical exercise ameliorated these negative effects, as evidenced by significantly lower phosphorylation levels of ERK and JNK (*P* < 0.05 and *P* < 0.05; Figures 6A–D). These findings showed that physical exercise can modulate the expression of MAPKs signaling cascades especially ERK1/2 and JNK pathways.

## Discussion

Physical exercise has beneficial effects on both peripheral tissues and CNS, and is a kind of safe and effective intervention to improve cognitive function (Maugeri et al., 2021). In human and animal models, the multiple benefits of regular physical exercise have been fully demonstrated. Whether aerobic exercise, anaerobic exercise or resistance exercise, are all considered to be beneficial to physical and mental health (Xu et al., 2021). More and more evidence showed that physical exercise can help maintain optimal cerebrovascular function, thereby preventing or slowing down the occurrence and development of cognitive impairment (Bliss et al., 2021). In this study, we found that wheel running exercise for 2 months can improve the spatial memory and non-spatial memory of CCH rats (Figures 1B, 2A–E). This is consistent with our previous study, which found that physical exercise alleviated the cognitive impairment of 2VO rats, and the effects tended to be more pronounced as the duration of exercise increased (Jiang et al., 2021). Similarly, some studies have shown that long-term physical exercise has positive effects through delaying the onset of physiological memory loss. However, it is worth mentioning that the late-onset exercise intervention has also shown positive impacts in delaying brain aging (De la Rosa et al., 2020). At present, the effects of pharmacological treatment for patients with cognitive impairment and dementia are limited, which not only brings a huge economic burden to society, but also seriously affects the quality of life of families facing such conditions. Since physical exercise has significant beneficial effects on the cognitive function of different diseases, it has aroused a lot of research interests as a kind of safe and effective non-drug treatment (Chan et al., 2021; Consorti et al., 2021). Physical exercise cannot only be used as a therapeutic protocol for neurological diseases, but also help to unlock the potential molecular targets of pharmacological methods (Consorti et al., 2021). Recently, exercise mimetics have been proposed as a class of therapeutics that can specifically mimic or enhance the therapeutic effects of physical exercise. Clarifying the mechanism mediating the beneficial impacts of exercise at the molecular, cellular and system levels is essential for the development of novel exercise mimetics (Gubert and Hannan, 2021). However, the underlying mechanism by which exercise may improve cognition remains unclear.

Recently, more and more studies have shown that the changes in astrocytes may be a key mechanism accounting for the improvement of cognitive and executive functions related to exercise (Li et al., 2021; Maugeri et al., 2021). Astrocytes are the main homeostasis and defense elements of the brain, helping to maintain cognitive reserve through a variety of mechanisms (Verkhratsky et al., 2021). They can communicate with neurons and other astrocytes in a bidirectional manner by releasing transmitters, increasing synaptic activity and strength, and play an active role in storing and processing information in the brain (Maugeri et al., 2021). It has been shown that the multiple effects of physical exercise on astrocytes include the number of new astrocytes increase, the basal level of catecholamines maintenance, the glutamate uptake enhance, the trophic factors release, and the better astrocytic coverage of cerebral blood vessels (Maugeri et al., 2021). In this study, we found that the astrocytes were activated in the brain of rats 2 months after 2VO, and they were mainly polarized toward the neurotoxic A1 phenotype. While physical exercise can switch the polarization of astrocytes from neurotoxic A1 to neuroprotective A2 phenotype (Figures 3A–C, 4A–C). Our previous study also reported that physical exercise cannot only modulate the transition of microglia from M1 to M2 phenotype, but also regulate the phenotypic polarization of astrocytes (Jiang et al., 2021). Consistently, similar studies have revealed that the low intensity motor balance and coordinated exercise can suppress the phenotypic polarization of M1 microglia and A1 astrocytes in the hippocampus of AD mice, contributing to the inhibition of Aβ accumulation and counteraction of behavioral and cognitive decline (Nakanishi et al., 2021). Recent studies have also emphasized the importance of physical activity as an intervention to prevent development of a severe form of COVID-19, as exercise can suppress excessive immune responses and trigger anti-inflammatory functions to promote psychological health (Maugeri et al., 2021). Interestingly, studies have shown that astrocytes may be the perspective targets of exercise mimetics (Lalo and Pankratov, 2021). In general, astrocytes are the central components for physical exercise-induced cognitive improvement in many neurodegenerative and neurovascular diseases (Li et al., 2021). The modulation of astrocytes polarization from neurotoxic A1 to neuroprotective A2 phenotypes may be an important mechanism involved.

As mentioned before, studies have indicated that primary cilia are involved in the regulation of the A1/A2 phenotypic polarization of astrocytes. It is well known that primary cilia can regulate astrocyte proliferation, polarization and tissue regeneration (Sterpka et al., 2020). Since primary cilia originate from the maternal centrioles, astrocytes proliferation must be coordinated with the dismantling and ciliogenesis of astrocytic cilia (Sterpka and Chen, 2018). Studying astrocytic primary cilia will provide useful clues for intervention in reactive astrocytes to combat various neuropathologies such as brain injury and ischemia (Sterpka and Chen, 2018). Primary cilia can be used as sensors for chemical and mechanical cues from cellular environment and play critical roles in transducing environmental stimuli and concentrating signal molecules to regulate cellular properties (Sun et al., 2021; Tereshko et al., 2021). However, current research about the effects of physical exercise on primary cilia is still limited. In this study, we found that the average length of primary cilia in the corpus callosum varied between 2.62 ± 0.33 μm in the sham group. Compared with the sham group, the length of astrocytic primary cilia in rats with CCH was shortened. In addition, the incidence of cilia also showed a decreasing trend, but there was no significant difference. However, physical exercise can significantly increase the expression and length of astrocytic primary cilia in the brain of rats 2 months after 2VO (Figures 5A–C). The length of primary cilia is closely related to their functions. Studies have found that primary cilia in hypocretin/orexin cells of obese mice were shorter, resulting in losing their ability to sense the local environment in the lateral/perifonical hypothalamus (Tan et al., 2020). Similarly, the shortened Arl13b-positive astrocytic cilia can be observed in the hippocampus of mice with spontaneous seizures (Sterpka et al., 2020). Whereas the ciliary length elongation has been proposed to fine-tune signaling activity of organelles in response to changes in the extracellular environment. The longer the cilia, the more functional they are and the greater capacity for signaling transduction in their internal receptor system (Brodsky et al., 2017; Sterpka et al., 2020). Therefore, astrocytic primary cilia are shortened after CCH, which may impair the ability of astrocytes to maintain normal function and perceive local damage environment, leading astrocytes polarization primarily toward the neurotoxic A1 phenotype. In contrast, physical exercise can rescue this negative impact and modulate astrocytes polarization to neuroprotective A2 phenotype by promoting primary cilia expression and elongation.

In order to clarify the possible mechanisms involved in the regulation of primary ciliogenesis and astrocyte polarization by physical exercise, we further observed the expression of mitogen-activated protein kinases (MAPKs) cascades. The MAPKs cascades mainly include JNK, P38, and ERK signaling pathways. They are involved in the regulation of cell proliferation, differentiation, migration, apoptosis and senescence, and are core components in signaling networks in various mammalian cells (Asih et al., 2020). Additionally, MAPKs are also the main effectors of inflammatory and mechanical stress (He et al., 2016). In this study, we found that compared with the sham group, the ERK and JNK signaling pathways in the brain of rats with CCH were activated, and physical exercise significantly reduced the expression of phosphorylated ERK and JNK, but without significant effects on the expression of the phosphorylated P38 (Figures 6A–D). These results are consistent with our previous study. We previously revealed that physical exercise can reduce ERK and JNK phosphorylation in the brain of rats 28 days after 2VO and promote microglia polarization from neurotoxic M1 to neuroprotective M2 phenotype, and finally promote remyelination and cognition improvement (Jiang et al., 2021). These findings suggest that the ERK and JNK signaling pathways may be the important effectors of exercise-induced anti neuroinflammatory responses. The inhibition of MAPKs activation, such as ERK, JNK, or P38, cannot only reduce inflammatory factors released from astrocytes and prevent astrocytes forming neurotoxic activity, but also increase neurotrophic factors release, maintain stable expression of glutamine synthetase from astrocytes, and promote neuroprotective effects of astrocytes (Efremova et al., 2017; Li et al., 2017; Liu et al., 2017; Wang et al., 2018, 2021; Zhou et al., 2019). Studies have also shown that mechanical signals sensed by primary cilia were transduced into biochemical signals involving pathways including ERK, JNK, and P38 (He et al., 2016). In some ciliopathy and cystic disease, MAPKs are important effector pathways closely associated with the process of ciliogenesis (Ma et al., 2013). Therefore, physical exercise may reduce the activation of ERK and JNK signaling pathways through regulating astrocytic primary cilia, and promote the activated astrocytes polarization from neurotoxic A1 to A2 phenotype to exert neuroprotective effects.

**FIGURE 7:**
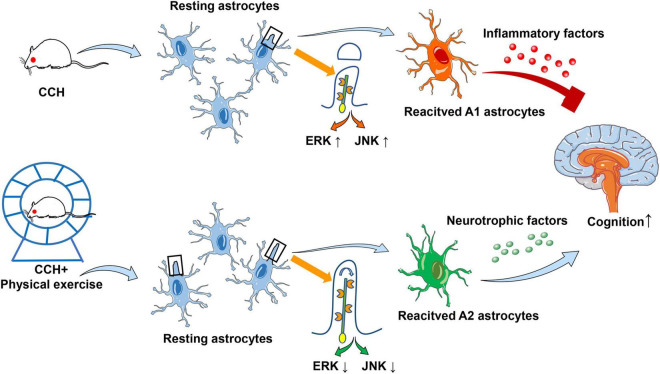
Schematic diagram for the possible mechanism of primary cilia and astrocyte polarization involved in physical exercise induced cognitive improvement in rats with chronic cerebral perfusion.

In summary, we have provided new evidence for clarifying the mechanism of physical exercise induced cognitive function recovery in CCH. Physical exercise can promote reactive astrocytes to exert neuroprotective roles by enhancing primary cilia length and ciliogenesis, and finally improve the cognitive function of rats with CCH. Moreover, the JNK and ERK signaling pathways are at least partly involved in this regulatory process (Figure 7).

## Data Availability Statement

The original contributions presented in the study are included in the article/supplementary material, further inquiries can be directed to the corresponding authors.

## Ethics Statement

The animal study was reviewed and approved by Institutional Animal Care and Use Committee of Wuhan University Center for Animal Experiment.

## Author Contributions

TJ and WJL conceived and designed the experiments. WYC, JYL, and BRW performed research and data acquisition. JBL and WYC analyzed and interpreted data. TJ and WX wrote the manuscript. WJL, WX, and JBL reviewed and edited the manuscript. All authors read the submitted version of this article and approved it for publication.

## Conflict of Interest

The authors declare that the research was conducted in the absence of any commercial or financial relationships that could be construed as a potential conflict of interest.

## Publisher’s Note

All claims expressed in this article are solely those of the authors and do not necessarily represent those of their affiliated organizations, or those of the publisher, the editors and the reviewers. Any product that may be evaluated in this article, or claim that may be made by its manufacturer, is not guaranteed or endorsed by the publisher.

## References

[B1] Alhassen W. Chen S. Vawter M. Robbins B. K. Nguyen H. Myint T. N. (2021). Patterns of cilia gene dysregulations in major psychiatric disorders. *Prog. Neuropsychopharmacol. Biol. Psychiatry* 109:110255. 10.1016/j.pnpbp.2021.110255 33508383 PMC9121176

[B2] Alvarez-Satta M. Moreno-Cugnon L. Matheu A. (2019). Primary cilium and brain aging: role in neural stem cells, neurodegenerative diseases and glioblastoma. *Ageing Res. Rev.* 52 53–63. 10.1016/j.arr.2019.04.004 31004829

[B3] Asih P. R. Prikas E. Stefanoska K. Tan A. R. P. Ahel H. I. Ittner A. (2020). Functions of p38 MAP kinases in the central nervous system. *Front. Mol. Neurosci.* 13:570586. 10.3389/fnmol.2020.570586 33013322 PMC7509416

[B4] Bliss E. S. Wong R. H. Howe P. R. Mills D. E. (2021). Benefits of exercise training on cerebrovascular and cognitive function in ageing. *J. Cereb. Blood Flow Metab.* 41 447–470. 10.1177/0271678X20957807 32954902 PMC7907999

[B5] Brach T. L. Gaitan J. M. Okonkwo O. C. (2021). Effect of aerobic exercise training on mood and cognition in adults at risk for Alzheimer’s disease. *Alzheimers Dement.* 17(Suppl. 2):e058523. 10.1002/alz.058523

[B6] Brodsky M. Lesiak A. J. Croicu A. Cohenca N. Sullivan J. M. Neumaier J. F. (2017). 5-HT6 receptor blockade regulates primary cilia morphology in striatal neurons. *Brain Res.* 1660 10–19. 10.1016/j.brainres.2017.01.010 28087224 PMC5392252

[B7] Chan W. C. Lee A. T. C. Lam L. C. W. (2021). Exercise for the prevention and treatment of neurocognitive disorders: new evidence and clinical recommendations. *Curr. Opin. Psychiatry* 34 136–141. 10.1097/YCO.0000000000000678 33470667

[B8] Chow Z. S. Moreland A. T. Macpherson H. Teo W. P. (2021). The central mechanisms of resistance training and its effects on cognitive function. *Sports Med.* 51 2483–2506. 10.1007/s40279-021-01535-5 34417978

[B9] Consorti A. Di Marco I. Sansevero G. (2021). Physical exercise modulates brain physiology through a network of long- and short-range cellular interactions. *Front. Mol. Neurosci.* 14:710303. 10.3389/fnmol.2021.710303 34489641 PMC8417110

[B10] De la Rosa A. Olaso-Gonzalez G. Arc-Chagnaud C. Millan F. Salvador-Pascual A. Garcia-Lucerga C. (2020). Physical exercise in the prevention and treatment of Alzheimer’s disease. *J. Sport Health Sci.* 9 394–404. 10.1016/j.jshs.2020.01.004 32780691 PMC7498620

[B11] Du Y. Song Y. Zhang X. Luo Y. Zou W. Zhang J. (2020). Leptin receptor deficiency protects mice against chronic cerebral hypoperfusion-induced neuroinflammation and white matter lesions. *Mediators Inflamm.* 2020:7974537. 10.1155/2020/7974537 33380900 PMC7762643

[B12] Duncombe J. Kitamura A. Hase Y. Ihara M. Kalaria R. N. Horsburgh K. (2017). Chronic cerebral hypoperfusion: a key mechanism leading to vascular cognitive impairment and dementia. Closing the translational gap between rodent models and human vascular cognitive impairment and dementia. *Clin. Sci. (Lond)* 131 2451–2468. 10.1042/CS20160727 28963120

[B13] Efremova L. Chovancova P. Adam M. Gutbier S. Schildknecht S. Leist M. (2017). Switching from astrocytic neuroprotection to neurodegeneration by cytokine stimulation. *Arch. Toxicol.* 91 231–246. 10.1007/s00204-016-1702-2 27052459

[B14] Feng T. Yamashita T. Sasaki R. Tadokoro K. Matsumoto N. Hishikawa N. (2021). Protective effects of edaravone on white matter pathology in a novel mouse model of Alzheimer’s disease with chronic cerebral hypoperfusion. *J. Cereb. Blood Flow Metab.* 41 1437–1448. 10.1177/0271678X20968927 33106078 PMC8142121

[B15] Ghafarimoghadam M. Mashayekh R. Gholami M. Fereydani P. Shelley-Tremblay J. Kandezi N. (2022). A review of behavioral methods for the evaluation of cognitive performance in animal models: current techniques and links to human cognition. *Physiol. Behav.* 244:113652. 10.1016/j.physbeh.2021.113652 34801559

[B16] Gubert C. Hannan A. J. (2021). Exercise mimetics: harnessing the therapeutic effects of physical activity. *Nat. Rev. Drug Discov.* 20 862–879. 10.1038/s41573-021-00217-1 34103713

[B17] He Z. Leong D. J. Zhuo Z. Majeska R. J. Cardoso L. Spray D. C. (2016). Strain-induced mechanotransduction through primary cilia, extracellular ATP, purinergic calcium signaling, and ERK1/2 transactivates CITED2 and downregulates MMP-1 and MMP-13 gene expression in chondrocytes. *Osteoarthritis Cartilage* 24 892–901. 10.1016/j.joca.2015.11.015 26687824

[B18] Hu L. Wang B. Zhang Y. (2017). Serotonin 5-HT6 receptors affect cognition in a mouse model of Alzheimer’s disease by regulating cilia function. *Alzheimers Res. Ther.* 9:76. 10.1186/s13195-017-0304-4 28931427 PMC5607612

[B19] Jiang T. Luo J. Pan X. Zheng H. Yang H. Zhang L. (2021). Physical exercise modulates the astrocytes polarization, promotes myelin debris clearance and remyelination in chronic cerebral hypoperfusion rats. *Life Sci.* 278:119526. 10.1016/j.lfs.2021.119526 33894268

[B20] Jiang T. Zhang L. Pan X. Zheng H. Chen X. Li L. (2017). Physical exercise improves cognitive function together with microglia phenotype modulation and remyelination in chronic cerebral hypoperfusion. *Front. Cell Neurosci.* 11:404. 10.3389/fncel.2017.00404 29311834 PMC5743796

[B21] Kim J. H. Ko P. W. Lee H. W. Jeong J. Y. Lee M. G. Kim J. H. (2017). Astrocyte-derived lipocalin-2 mediates hippocampal damage and cognitive deficits in experimental models of vascular dementia. *Glia* 65 1471–1490. 10.1002/glia.23174 28581123

[B22] Lalo U. Pankratov Y. (2021). Astrocytes as perspective targets of exercise- and caloric restriction-mimetics. *Neurochem. Res.* 46 2746–2759. 10.1007/s11064-021-03277-2 33677759 PMC8437875

[B23] Leardini-Tristao M. Andrade G. Garcia C. Reis P. A. Lourenco M. Moreira E. T. S. (2020). Physical exercise promotes astrocyte coverage of microvessels in a model of chronic cerebral hypoperfusion. *J. Neuroinflamm.* 17:117. 10.1186/s12974-020-01771-y 32299450 PMC7161182

[B24] Li D. Liu N. Zhao H. H. Zhang X. Kawano H. Liu L. (2017). Interactions between Sirt1 and MAPKs regulate astrocyte activation induced by brain injury in vitro and in vivo. *J. Neuroinflamm.* 14:67. 10.1186/s12974-017-0841-6 28356158 PMC5372348

[B25] Li F. Geng X. Yun H. J. Haddad Y. Chen Y. Ding Y. (2021). Neuroplastic effect of exercise through astrocytes activation and cellular crosstalk. *Aging Dis.* 12 1644–1657. 10.14336/AD.2021.0325 34631212 PMC8460294

[B26] Liddelow S. A. Guttenplan K. A. Clarke L. E. Bennett F. C. Bohlen C. J. Schirmer L. (2017). Neurotoxic reactive astrocytes are induced by activated microglia. *Nature* 541 481–487. 10.1038/nature21029 28099414 PMC5404890

[B27] Liu D. Zhang M. Rong X. Li J. Wang X. (2017). Potassium 2-(1-hydroxypentyl)-benzoate attenuates neuronal apoptosis in neuron-astrocyte co-culture system through neurotrophy and neuroinflammation pathway. *Acta Pharm. Sin. B* 7 554–563. 10.1016/j.apsb.2017.06.006 28924549 PMC5595293

[B28] Ma M. Tian X. Igarashi P. Pazour G. J. Somlo S. (2013). Loss of cilia suppresses cyst growth in genetic models of autosomal dominant polycystic kidney disease. *Nat. Genet.* 45 1004–1012. 10.1038/ng.2715 23892607 PMC3758452

[B29] Marques-Aleixo I. Beleza J. Sampaio A. Stevanovic J. Coxito P. Goncalves I. (2021). Preventive and therapeutic potential of physical exercise in neurodegenerative diseases. *Antioxid Redox Signal.* 34 674–693. 10.1089/ars.2020.8075 32159378

[B30] Maugeri G. D’Agata V. Magri B. Roggio F. Castorina A. Ravalli S. (2021). Neuroprotective effects of physical activity *via* the adaptation of astrocytes. *Cells* 10:1542. 10.3390/cells10061542 34207393 PMC8234474

[B31] Miyamoto N. Magami S. Inaba T. Ueno Y. Hira K. Kijima C. (2020). The effects of A1/A2 astrocytes on oligodendrocyte linage cells against white matter injury under prolonged cerebral hypoperfusion. *Glia* 68 1910–1924. 10.1002/glia.23814 32108971

[B32] Nakanishi K. Sakakima H. Norimatsu K. Otsuka S. Takada S. Tani A. (2021). Effect of low-intensity motor balance and coordination exercise on cognitive functions, hippocampal Abeta deposition, neuronal loss, neuroinflammation, and oxidative stress in a mouse model of Alzheimer’s disease. *Exp. Neurol.* 337:113590. 10.1016/j.expneurol.2020.113590 33388314

[B33] Padilha C. S. Figueiredo C. Minuzzi L. G. Chimin P. Deminice R. Kruger K. (2021). Immunometabolic responses according to physical fitness status and lifelong exercise during aging: new roads for exercise immunology. *Ageing Res. Rev.* 68:101341. 10.1016/j.arr.2021.101341 33839332

[B34] Payan-Gomez C. Rodriguez D. Amador-Munoz D. Ramirez-Clavijo S. (2018). Integrative analysis of global gene expression identifies opposite patterns of reactive astrogliosis in aged human prefrontal cortex. *Brain Sci.* 8:227. 10.3390/brainsci8120227 30572619 PMC6317157

[B35] Presa J. L. Saravia F. Bagi Z. Filosa J. A. (2020). Vasculo-neuronal coupling and neurovascular coupling at the neurovascular unit: impact of hypertension. *Front. Physiol.* 11:584135. 10.3389/fphys.2020.584135 33101063 PMC7546852

[B36] Pronk N. P. (2021). Neuroplasticity and the role of exercise and diet on cognition. *Am. J. Clin. Nutr.* 113 1392–1393. 10.1093/ajcn/nqab083 33851206

[B37] Scheffer D. D. L. Latini A. (2020). Exercise-induced immune system response: anti-inflammatory status on peripheral and central organs. *Biochim. Biophys. Acta Mol. Basis Dis.* 1866:165823. 10.1016/j.bbadis.2020.165823 32360589 PMC7188661

[B38] Singh M. Garrison J. E. Wang K. Sheffield V. C. (2019). Absence of BBSome function leads to astrocyte reactivity in the brain. *Mol. Brain* 12:48. 10.1186/s13041-019-0466-z 31072410 PMC6509862

[B39] Sipos E. Komoly S. Acs P. (2018). Quantitative comparison of primary cilia marker expression and length in the mouse brain. *J. Mol. Neurosci.* 64 397–409. 10.1007/s12031-018-1036-z 29464516

[B40] Sterpka A. Chen X. (2018). Neuronal and astrocytic primary cilia in the mature brain. *Pharmacol. Res.* 137 114–121. 10.1016/j.phrs.2018.10.002 30291873 PMC6410375

[B41] Sterpka A. Yang J. Strobel M. Zhou Y. Pauplis C. Chen X. (2020). Diverged morphology changes of astrocytic and neuronal primary cilia under reactive insults. *Mol. Brain* 13:28. 10.1186/s13041-020-00571-y 32122360 PMC7053156

[B42] Sun Z. Wang B. Chen C. Li C. Zhang Y. (2021). 5-HT6R null mutatrion induces synaptic and cognitive defects. *Aging Cell* 20:e13369. 10.1111/acel.13369 33960602 PMC8208783

[B43] Tan Y. Hang F. Liu Z. W. Stoiljkovic M. Wu M. Tu Y. (2020). Impaired hypocretin/orexin system alters responses to salient stimuli in obese male mice. *J. Clin. Invest.* 130 4985–4998. 10.1172/JCI130889 32516139 PMC7456212

[B44] Tang E. Y. H. Robinson L. Price C. (2021). Stroke: time to address cognition. *Br. J. Gen. Pract.* 71 104–105. 10.3399/bjgp21X714977 33632680 PMC7909917

[B45] Tang H. Gao Y. Zhang Q. Nie K. Zhu R. Gao L. (2017). Chronic cerebral hypoperfusion independently exacerbates cognitive impairment within the pathopoiesis of Parkinson’s disease *via* microvascular pathologys. *Behav. Brain Res.* 333 286–294. 10.1016/j.bbr.2017.05.061 28578987

[B46] Tereshko L. Gao Y. Cary B. A. Turrigiano G. G. Sengupta P. (2021). Ciliary neuropeptidergic signaling dynamically regulates excitatory synapses in postnatal neocortical pyramidal neurons. *eLife* 10 e65427. 10.7554/eLife.65427 33650969 PMC7952091

[B47] Thammisetty S. S. Renaud L. Picher-Martel V. Weng Y. C. Calon F. Saikali S. (2021). Targeting TDP-43 pathology alleviates cognitive and motor deficits caused by chronic cerebral hypoperfusion. *Neurotherapeutics* 18 1095–1112. 10.1007/s13311-021-01015-8 33786804 PMC8423945

[B48] Ungvari Z. Toth P. Tarantini S. Prodan C. I. Sorond F. Merkely B. (2021). Hypertension-induced cognitive impairment: from pathophysiology to public health. *Nat. Rev. Nephrol.* 17 639–654. 10.1038/s41581-021-00430-6 34127835 PMC8202227

[B49] Verkhratsky A. Augusto-Oliveira M. Pivoriunas A. Popov A. Brazhe A. Semyanov A. (2021). Astroglial asthenia and loss of function, rather than reactivity, contribute to the ageing of the brain. *Pflugers. Arch.* 473 753–774. 10.1007/s00424-020-02465-3 32979108

[B50] Wang S. Zhang H. Geng B. Xie Q. Li W. Deng Y. (2018). 2-arachidonyl glycerol modulates astrocytic glutamine synthetase *via* p38 and ERK1/2 pathways. *J. Neuroinflamm.* 15 220. 10.1186/s12974-018-1254-x 30075820 PMC6091076

[B51] Wang T. Sun Q. Yang J. Wang G. Zhao F. Chen Y. (2021). Reactive astrocytes induced by 2-chloroethanol modulate microglia polarization through IL-1beta, TNF-alpha, and iNOS upregulation. *Food Chem. Toxicol.* 157:112550. 10.1016/j.fct.2021.112550 34517076

[B52] Xie Y. C. Yao Z. H. Yao X. L. Pan J. Z. Zhang S. F. Zhang Y. (2018). Glucagon-like peptide-2 receptor is involved in spatial cognitive dysfunction in rats after chronic cerebral hypoperfusion. *J Alzheimers Dis.* 66 1559–1576. 10.3233/JAD-180782 30452417

[B53] Xu M. Zhu J. Liu X. D. Luo M. Y. Xu N. J. (2021). Roles of physical exercise in neurodegeneration: reversal of epigenetic clock. *Transl. Neurodegener.* 10:30. 10.1186/s40035-021-00254-1 34389067 PMC8361623

[B54] Xu Y. Zhang S. Sun Q. Wang X. Q. Chai Y. N. Mishra C. (2020). Cholinergic dysfunction involvement in chronic cerebral hypoperfusion-induced impairment of medial Septum-dCA1 neurocircuit in rats. *Front. Cell Neurosci.* 14:586591. 10.3389/fncel.2020.586591 33132852 PMC7550820

[B55] Zhou F. Liu X. Gao L. Zhou X. Cao Q. Niu L. (2019). HIV-1 Tat enhances purinergic P2Y4 receptor signaling to mediate inflammatory cytokine production and neuronal damage *via* PI3K/Akt and ERK MAPK pathways. *J. Neuroinflamm.* 16:71. 10.1186/s12974-019-1466-8 30947729 PMC6449963

